# Advancing transdermal drug delivery through 4D bioprinting and dynamic skin modelling

**DOI:** 10.3389/fddev.2026.1753384

**Published:** 2026-02-03

**Authors:** Prina Mehta

**Affiliations:** Leicester School of Pharmacy, De Montfort University, Leicester, United Kingdom

**Keywords:** 4D printing, bioprinting, skin-on-a-chip, smart biomaterials, stimuli-responsive materials, transdermal delivery

## Abstract

Transdermal drug delivery (TDD) provides a non-invasive approach for sustained drug release. However, traditional models present limitations in capturing the dynamic interactions between drugs, skin and the environmental factors over time. The incorporation of time as a critical dimension alongside three-dimensional (3D) structures in four-dimensional (4D) modelling offers a promising solution by simulating the temporal evolution of drug diffusion and skin responses. In this review, 4D modelling refers to the computational and material-based systems that incorporate time-dependent changes whereas 4D bioprinting specifically involves fabrication of dynamic, stimuli-responsive skin constructs. Together, these approaches create temporally adaptive models which are ideal for simulating drug permeation and skin behaviour. This review will explore the potential application of 4D modelling in TDD, primarily focusing on and emphasising its capacity to predict drug permeation, release kinetics and skin interactions in response to variables such as hydration, temperature and mechanical impact. 4D bioprinting provides a more accurate depiction of real-world scenarios, enabling researchers to optimise drug formulations whilst minimising reliance on empirical testing. Despite challenges associated with cost and complexity, 4D modelling presents considerable opportunities, particularly in the advancement of personalised medicine. The integration of artificial intelligence could further enhance these models, resulting in more accurate predictions. By addressing both spatial and temporal dimensions, 4D constructs will continue to evolve and have the potential to transform TDD; particularly in the context of individualised treatment where dynamic patient-specific variables can be integrated to develop more effective and tailored treatments.

## 1 Introduction

Developments in technology and material science has transformed existing drug delivery methods, eliminating the limitations of traditional approaches. Transdermal Drug Delivery (TDD) is already an established route of administration that enables systemic or local drug delivery through the skin and TDD has steadily gained traction in drug delivery research due to being non-invasive, achieving various active-release kinetics and by-passing first-pass metabolism. Compared to existing routes of administration such as oral and parenteral approaches, TDD provides improved patient compliance particularly for long-term therapies. Despite these benefits, TDD presents some challenges including limitations in drug penetration, irritation and unpredictable interactions with the skins’ dynamic properties and this in turn has implications in its application (Vaseem et al., 2024).

Traditional 2D and 3D models have been extensively used to study these challenges alongside skin-drug interactions (Wang et al., 2021). While these models have significantly contributed to our understanding of the complexity of the skin, one of the biggest limitations is the inability to fully capture the dynamic nature of skin. The ever-changing barrier of human skin is continuously experiencing physiological changes which are influenced by a multitude of factors such as hydration levels, intrinsic ageing, and exposure to environmental stressors (Joshi et al., 2020; Ramezani and Mohd Ripin, 2023). This non-exhaustive list of factors plays a critical role in the efficiency of drug permeation through the skin to reach the targeted tissue. For example, dehydrated skin or a loss in moisture can result in a decrease in drug permeability in turn hindering absorption of therapeutic actives (Brito et al., 2024; Lee et al., 2023; Tapfumaneyi et al., 2025). Similarly, intrinsic and actinic ageing can modify lipid composition, collagen density and barrier function of the skin, affecting drug diffusion rate across the skin (Lee et al., 2023). In addition to this, factors and stresses such as exposure to UV radiation, air pollution and to an extent circadian rhythms can trigger biochemical-based responses which can affect skin permeability. It is these real-time physiological adaptations that 2D and 3D models fail to replicate and therefore cannot accurately represent behaviour of formulations when applied to actual human skin under various conditions.

This gap in modelling highlights the need for more advanced biomimetic approaches to enhance simulation of the constant-changing characteristics of the skin to enhance the precision of TDD studies. The emergence of 4D skin models is a ground-breaking advancement, which encompasses the study of skin biology and drug delivery (Aftab et al., 2025; Alanazi et al., 2025; Gazzaniga et al., 2023; Liu et al., 2015; Vehse et al., 2014; Wang et al., 2018; Zu et al., 2022). As shown in Table 1, where traditional 2D and 3D models focus predominantly on static or structurally complex representations of skin, programmable 4D constructs integrate the dimension of time and smart materials, offering dynamic adaptability. This introduction of temporal responsiveness allows scientists to observe and analyse dynamic processes like ageing, wound healing and reparation of barrier function in ways that previously have not been possible and can therefore provide deeper insights into drug diffusion rates, retention and overall efficacy (Joshi et al., 2020; Martinez-De-Anda et al., 2023). By capturing these temporal changes, 4D scaffolds portray a much more accurate simulation of how human skin behaves in real-word conditions (Martinez-De-Anda et al., 2023).

**TABLE 1:** Comparison of key features, materials and applications of 2D, 3D and 4D printing in transdermal drug delivery.

Aspect	2D printing	3D printing	4D printing
Definition	Deposition of drug-loaded inks onto flat substrates	Additive manufacturing of solid dosage forms, layer by layer	3D printing integrating a temporal component and smart materials that respond to various stimuli
Process Parameters	- Print speed - Resolution (dpi) - Ink viscosity - Substrate type	- Layer thickness - Print speed - Extrusion temperature - Bed temperature - Infill density	- Layer thickness - Print speed - Extrusion temperature - Bed temperature - Infill density - Stimulus trigger (pH, temperature, moisture) - Shape-memory programming
Materials	- Drug-loaded inks - Edible polymer films - Solvents	- Thermoplastics (PLA, PVA) - Hydrogels - Biodegradable polymers - Resins	- Smart polymers (shape memory) - Hydrogels - Stimuli-responsive composites
Advantages	✓ High dose precision ✓ Rapid onset ✓ Low cost	✓ Controlled and multiphase release ✓ Complex geometries ✓ Personalised dosing ✓ Ability to integrate microneedles	✓ Dynamic response to environment ✓ Targeted and localised therapy ✓ Adaptive treatment
Limitations	✗ Limited drug loading ✗ Restricted to thin films ✗ Mostly single phase	✗ Slower production ✗ High costs ✗ Regulatory hurdles	✗ Cost implications ✗ Limited material availability ✗ Complex validation
Transdermal Drug Delivery Examples	- Printed transdermal patches for nicotine and hormones - Buccal or dermal films loaded with NSAIDs	- 3D-printed microneedle arrays for various applications, including insulin delivery, anticancer therapy and vaccines - Hydrogel-based wound dressings with antibiotics	- “Smart” shape-changing matrices due to various chemical and physical stimuli - Skin-on-a-chip devices
References	- (Bal et al., 2025; Eleftheriadis et al., 2020; Kurz et al., 2009; Maver et al., 2019; Swaminathan et al., 2020)	- (Aldawood et al., 2024; Algellay et al., 2023; Chachlioutaki et al., 2020; Chen X. D. et al., 2024; Muldoon et al., 2022; Nail et al., 2025; Prabhu et al., 2025; Prajapat et al., 2025; Zhang et al., 2024; Zhang and Wang, 2022)	- (Barros et al., 2025; Che et al., 2024; Chen et al., 2012; Cho et al., 2024; Huang et al., 2024; Mohamadali et al., 2023; Naeem et al., 2025; Sadraei et al., 2025; Sadraei and Naghib, 2025; Wufuer et al., 2016; Zoio and Oliva, 2022)

Unlike previous reviews that focus primarily on materials or printing techniques, this mini-review will show how 4D bioprinting and dynamic skin models directly advance TDD applications. The impact of these on various parameters such as release kinetics and patient-specific modelling will be evaluated while identifying current gaps in translation and proposing future research priorities.

## 2 Advances in 4D bioprinted constructs for drug delivery applications

Conventional 2D and 3D skin models lack the capability to mimic temporal changes in barrier function, hydration, deformation and ageing. As a result, they cannot replicate dynamic drug diffusion pathways which in turn limits their predictive value in TDD research. Technological advances like 4D bioprinting can overcome these limitations.

**FIGURE 1:**
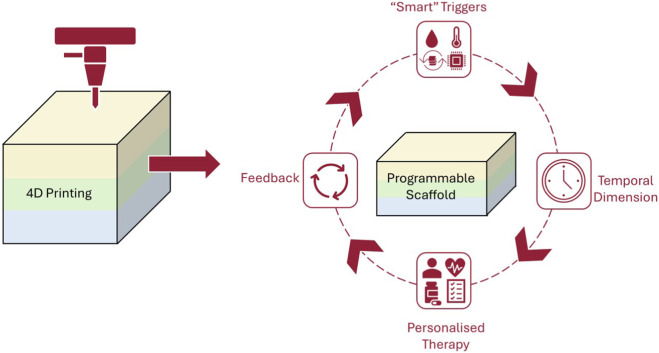
Conceptual schematic of 4D printing workflow, highlighting how programmable scaffolds respond to smart triggers and temporal changes to enable dynamic and personalised therapy with integral feedback mechanisms.

Bioprinting is an advanced manufacturing method that utilises bioinks, composed of living cells, biomaterials and growth factors, to fabricate tissue-like structures layer by layer (Mendoza-Cerezo et al., 2023; Tripathi et al., 2023). Unlike traditional printing, bioprinting aims to replicate the architecture and functionality of biological tissues, resulting in complex, stratified constructs such as skin equivalents (Dey and Ozbolat, 2020; Mendoza-Cerezo et al., 2023). As shown in Figure 1, 4D printing combines smart biomaterials and time-dependent triggers to create adaptive scaffolds for personalised models. Bioprinting also provides precise spatial control over cell placement and material composition, allowing researchers to design programmable scaffolds that mimic native skin properties. More importantly, these technological advances have direct relevance to TDD as by enabling controlled temporal changes in architecture, hydration and matrix mechanics, 4D constructs can replicate the evolving skin barrier more accurately than static 3D models. This improved biomimicry then provides opportunity to evaluate drug permeation and release behaviour under physiologically dynamic conditions, as opposed to relying solely on static end-point measurements (Ashammakhi et al., 2018; Chen J. et al., 2024; Faber et al., 2024; Noroozi et al., 2023; Shie et al., 2019).

### 2.1 Advances in 4D bioprinting techniques

#### 2.1.1 Printing methods and process control

The evolution of bioprinting methods has considerably helped to advance 4D skin models (Aftab et al., 2025; Ashammakhi et al., 2018; Damiati et al., 2025; Gao et al., 2016; Kalogeropoulou et al., 2024; Kuang et al., 2019; Morouço et al., 2020; Noroozi et al., 2023; Yarali et al., 2024). Extrusion-based bioprinting remains the most used technique in skin modelling, predominantly due to its ability to accurately deposit highly viscous cell-laden bioinks (Ahn et al., 2023; Ghosh et al., 2025; He et al., 2025; Perez-Valle et al., 2020; Rossi et al., 2024; Wang et al., 2024). Similarly, inkjet printing is a popular approach due to high resolution and fast processing speeds; often used for patterning smaller biomolecules and cells (Arshad et al., 2020; Che et al., 2024; Ng et al., 2017; Wang et al., 2022). For more complex cell layering and spatial cellular arrangements, laser-assisted bioprinting is preferred as it offers the advantage of precise cell deposition without nozzle clogging (Chang and Sun, 2023; Douillet et al., 2022; Ishack and Lipner, 2020). For example, Douillet et al. developed fibroblast-collagen lattices with the ability to modulate cell-generated tensions within the matrix (Douillet et al., 2022). The skin-equivalent scaffolds displayed both epidermal and dermal layers and remained viable for more than 40 days. The resulting printed construct initially presented as a soft cross-linked hydrogel scaffold but over time, the fibroblasts remodelled, contracting and reorganising the matrix. Using 4D laser-printing techniques enabled real-time monitoring of the hydrogel polymerisation and cell placement; converting a static pattern into a living tissue that adapts its mechanical properties and architecture.

Using such evolved techniques enables the development of stratified skin constructs that can change and evolve overtime whilst mimicking crucial skin structural components such as dermal-epidermal junctions and microvascular networks.

#### 2.1.2 Cellular integration and functionalisation

Arguably, the most crucial aspect of 4D modelling stems from the integration of significant cellular components such as keratinocytes and fibroblasts. By adding dynamic, time-dependent triggers that can be tuned to each patient’s cellular and extracellular matrix profile, the field of personalised medicine can be transformed (Zablotskii et al., 2016).

Cells like keratinocytes and fibroblasts are fundamental due to the role they play in epidermal and dermal function (Douillet et al., 2022). As the evolution in material science and processes continues, the inclusion of immune-based cellular components such as Langerhan cells and macrophages has enhanced the responsiveness of 4D models to stimuli related to inflammation and immunogenicity; a crucial output to evaluate with respect to drug irritation and allergenicity studies (Koti et al., 2019). By utilising patient-derived immunological components and by developing programmable scaffolds with specific stiffness, degradation rates and biochemical gradients, researchers can mimic the personalised biomechanical and signaling environment of individual skin. The ongoing integration of vascular and neuron-based elements represents the next stage toward fully functional skin equivalents.

### 2.2 Smart biomaterials and stimuli integration in 4D skin models

The use of advanced technologies has not been the only factor to contribute to the development of 4D models.

Unlike processes that build on static, 3D concepts to mimic the structural complexity of skin tissue, 4D bioprinting incorporates a temporal element to allow for the constant dynamic responses to external stimuli (Li et al., 2017). This innovative approach can enable skin constructs to replicate how the skin adapts to various environmental stimuli such as temperature, humidity, and mechanical impact (Kim et al., 2024; Murphy and Atala, 2014; Ramezani and Mohd Ripin, 2023; Tran et al., 2022). Whilst there has been great interest in this field, there has been limited pharmaceutics-based published works in this area (Firth et al., 2018; Trenfield et al., 2019).

#### 2.2.1 Material innovations driving 4D bioprinting

The core of 4D bioprinting focuses on the use of “smart” biomaterials capable of responding to various stimuli and allow for time-dependent responses in the printed constructs to be captured (Alanazi et al., 2025; Chen J. et al., 2024; Chu et al., 2020; Ding et al., 2025; Mahmoud and Schulz-Siegmund, 2023; Naeem et al., 2025; Tibbits, 2014; Wang et al., 2025; Wu et al., 2025).

Hydrogels have been widely used in drug delivery systems for many years due to their high-water content and biocompatibility (Ma et al., 2024; Mohammadzadeh et al., 2025; Qutub et al., 2025; Sadraei and Naghib, 2025; Toews et al., 2025; Zöller et al., 2025). The ability to also personalise or optimise the physical properties of these “smart” materials has also made them a popular drug delivery matrix (Dai et al., 2019; Naficy et al., 2017; Narupai et al., 2021; Sadraei et al., 2025; Wu et al., 2025; Zu et al., 2022). Hydrogels can be engineered to be triggered to swell, shrink and change shape in response to environmental changes such as pH (Li et al., 2016; Mirani et al., 2017; Vijayavenkataraman et al., 2018), temperature (Abdullah and Okay, 2023; Thakur et al., 2023) or even the presence of enzymes (Ashammakhi et al., 2018; Cui et al., 2019; Nadgorny et al., 2016; Sadraei et al., 2025; Shie et al., 2019; Tran et al., 2022).

Another classification of “smart” materials includes shape-memory Polymers (SMPs) (Wei et al., 2017). Following deformation due to exposure to specific triggers (e.g., temperature, pH), SMPs can revert to their original configuration, making these materials advantageous in skin modelling where controlled movement and structural transformation is essential to emulate physiological skin behaviours (Alam et al., 2023; Cerbe et al., 2024; Invernizzi et al., 2018; Kalogeropoulou et al., 2024; Liu et al., 2015; Narupai et al., 2021; Pisani et al., 2022; Zhang et al., 2019).

Che et al. developed an innovative approach to fabricating microneedles using hydroxybutyl methacrylated chitosan (HBC-MA) using 3D printing (Che et al., 2024). HBC-MA possesses dual temperature and photo-sensitive properties which enabled the microneedles to change dimensions when exposed to different temperatures in turn improving their mechanical strength. This will allow the chitosan-based device to load, deliver and sustain the release of small molecular drugs as well as penetrate soft tissue effectively. A novel system has also been designed that integrated pH-responsive porous “sensors” with gentamicin-loaded alginate stents (Mirani et al., 2017). This drug delivery platform enabled on demand antibiotic release where pH fluctuations triggered the release of gentamicin to target bacterial infections, demonstrating a promising approach for responsive and localised 4D-based drug delivery.

#### 2.2.2 Dynamic environmental triggers in 4D models

In addition to smart materials, biomechanical and biochemical triggers are also becoming increasingly common in driving development of 4D models (Alanazi et al., 2025; Cabane et al., 2012; Chen et al., 2012; Ge et al., 2014; Roy et al., 2010; Talebian et al., 2019; Yin et al., 2014). Properties such as tension, elasticity and viscoelasticity must be considered during drug delivery modelling to account for the mechanical compliance of human skin (Aizarna-Lopetegui et al., 2025; Sutterby et al., 2022). These variables determine how the skin barrier deforms and recovers following stretching or compression. 4D models have the ability to incorporate external mechanical cues such as cyclic stretching or pressure to reproduce the physical exertion acting on human skin. Kaiser et al. presented a pneumatically driven skin-on-a-chip platform which coupled automated media perfusion with cyclic uniaxial compression and stretching. The scaffolds here saw an increase in the proteins associated with cell junctions in epithelial tissues (Claudin-1 and desmoplakin 1 and 2) under mechanical stimulation; highlighting stronger tight-junction and desmosome formation (Kaiser et al., 2024). Being able to assess these variables can help evaluate drug penetration under movement or during mechanical stress; an important factor often overlooked in static 3D models.

Alongside this, biochemical gradients, growth factors and signalling molecules can help set up a bioactive environment by guiding cell differentiation and immune responses to support skin behaviour on a cellular level (Imrie and Jin, 2025; Lai et al., 2025; Lai et al., 2024; Mathur et al., 2025). By integrating these dynamic stimuli in 4D constructs, it allows for more accurate predictions of inflammation, wound healing and allergic reactions in a controlled setting (Alanazi et al., 2025; Talebian et al., 2019).

### 2.3 Applications of 4D skin models in drug delivery

From a drug delivery perspective, the incorporation of time-dependent material and cellular responses fundamentally alters release kinetics and permeation. Temporal shifts in scaffold porosity, hydration state and mechanical stress can influence the effective diffusion coefficient within the construct and can either accelerate or delay permeation. These 4D-mediated changes can aid researchers in reviewing how formulations behave under realistic fluctuations in skin barrier function for example, by assessing temperature-responsive diffusion or hydration dependent flux.

#### 2.3.1 Enhancing drug delivery and permeation studies

The accuracy and precision of studies involving drug permeation in transdermal research has significantly improved with the implementation of 4D skin models. Comparative studies between 4D and 2D and 3D models show that while the latter provide the foundational understandings of tissue-engineered skin constructs, they fail to take into consideration the temporal aspects required to fully simulate skin processes. Traditional 2D models cannot account for the restructure of the skin barrier following impairment whereas 4D models can actively replicate the disruptions in the skin barriers as well as the recovery phases which inherently increases the reliability of the resulting absorption data.

An example of using machine learning modelling is the development of thermo-responsive 4D constructs which were evaluated using paracetamol as a model drug (Suryavanshi et al., 2023). Physiochemical analysis of the constructs revealed high entrapment efficiency (98.11% ± 1.5%) with uniform drug distribution throughout the 4D-printed matrix. *In vitro* release studies demonstrated complete and rapid release of paracetamol, reaching nearly 100% within 4 h at physiological temperature.

#### 2.3.2 Dynamic modelling of skin conditions

The ability for 4D models to exhibit real-time dynamic changes including fluctuations in hydration, temperature and pH is a key strength of such models. For example, dynamic hydration modelling can allow researchers to investigate Transepidermal Water Loss (TEWL) and how moisture loss and/or accumulation affects drug flux through the stratum corneum (Kundu et al., 2025). In a similar context, thermo-responsive materials can also help to simulate skin responses to thermal changes such as heat exposure, allowing for the study of temperature-dependent drug permeation. This responsive nature in turn allows researchers to simulate pathophysiological conditions such as eczema and inflammation which would be limited in traditional static models (Wufuer et al., 2016).

The use of 4D skin models has also extended to personalised medicine. Kapoor et al. reviewed the use of traditional 3D printing technologies with the aim to develop customised drug delivery systems which tailored to individualised patient needs (Kapoor et al., 2025).

#### 2.3.3 Integration with microfluidic systems

More innovative techniques and methods have emerged in recent years to demonstrate the full potential of 4D systems in transdermal delivery. Integrated microfluidic systems and sensor-loaded scaffolds allow for real time monitoring of drug diffusion and skin metabolism (Zoio and Oliva, 2022). Wufuer et al. (2016) developed a microfluidic skin-on-a-chip model capable of reproducing critical physiological responses such as inflammation-induced permeability changes which allowed the continuous measurement of drug absorption without the need to extract tissue (Wufuer et al., 2016).

A pumpless microfluidic skin-on-a-chip system using a full-thickness Human Skin Equivalent (HSE) model has also been developed (Abaci et al., 2016). The ability for this approach to maintain skin barrier function for 3 weeks allowed comparative links to be made to *in vivo* skin permeation data and therefore make more accurate predictions of real time monitoring of drug diffusion. Similarly, Mohamadali et al. engineered a skin-on-a-chip pumpless microfluidic device with polydimethylsiloxane microchannels (Mohamadali et al., 2023). Tissue culture studies showed promising results with the microfluidic system enhancing viability of the full-thickness skin samples for at least 7 days under controlled conditions. The nature of microfluidic-based devices enables kinetic parameters such as lag time, peak flux and steady-state permeation to be measured accurately with non-invasive analysis.

The kinetic readouts generated on-chip are the most powerful and useful when paired with high-resoluton, non-invasive imaging that helps to close the loop between continuous measurements and structural validation. By coupling these microfluidic systems with advanced imaging technologies like confocal microscopy and optical coherence tomography (OCT), it also possible to visualise drug distribution *in situ* and tissue changes over time. Confocal laser scanning microscopy has already shown it can present high-resolution maps off fluorescent markers and marked formulations across skin layers which when coupled with microfluidic systems can enhance the accuracy of non-invasive collected data. Similarly, OCT offers non-destructive imaging of structural and functional changes during *in vitro* testing which can help determine chemical distribution in soft tissues ((Belsey et al., 2023).

Overall, the shift from static 2D/3D constructs to dynamic 4D skin models gives benefits across multiple drug delivery parameters. Temperature responsive systems demonstrate approximately 2-fold increase in drug flux with a controlled 10 °C rise in skin surface temperature, consistent with documented activation energies for stratum corneum diffusion (Chen J. et al., 2024; LaCount et al., 2020). Similarly, dynamic hydration modelling indicates that permeability increases sharply above 75% relative humidity, reflecting physiologically relevant shifts in barrier function (Oftadeh et al., 2023; Yu et al., 2026). Microfluidic 4D skin-on-chip systems provide real-time kinetic outputs in turn improving accuracy in lag time estimation and steady-state permeation which are not achievable in static diffusion cells (Mohamadali et al., 2023; Zuieva et al., 2022). These measurable differences underpin the translational value of 4D models for predicting drug diffusion under real-world dynamically evolving s.

In combination, dynamic modelling (to reproduce time-dependent changes in hydration, temperature and mechanics), microfluidic systems (to capture real-time permeation kinetics) and advanced imaging (to localise drug distribution and confirm structural changes *in-situ*) demonstrates a predictive, patient-focused platform for TDD optimisation, linking evolving skin physiology to quantified permeation metrics, enhancing formulation optimisation for TDD.

## 3 Future perspectives and conclusion

Whilst the advancements highlighted in this mini-review show promise, significant challenges still exist with regards to reproducing and more specifically standardising 4D models in transdermal drug delivery. Variability in bioprinting process parameters (such as printing pressure, bioink rheology, and cell-culture timing) present reproducibility hurdles that complicate cross-laboratory comparisons (Perez-Valle et al., 2020). Current assessments of 3D skin bioprinting scrutinises the techniques and protocols required for these processes and concerns are amplified when temporal parameters are introduced. The introduction of time as another dimension adds a layer of complexity that may hinder high-throughput screening and scalability too. Cho et al. emphasised that there is a specific need for robust development protocols and validation frameworks for collation of consistent, reliable data suggesting that integration with microfluidic technologies and sensor systems could enhance throughput and real-time monitoring (Cho et al., 2024).

4D modelling goes beyond technical evolution. By adding dynamic, patient-specific factors, these models can bridge the gap between laboratory studies and clinical testing. They reduce the need for animal experiments and support ethical standards under the NC3Rs (Replacement. Reduction and Refinement). As seen in this review, 4D systems outperform traditional 3D models and simulations by capturing both spatial and time-based changes, leading to more accurate predictions of drug absorption in real-world conditions. In an ever-evolving world of technology and with the rapid emergence of artificial intelligence, the TDD remit is being revolutionised using machine learning to predict drug release and behaviour and to develop personalised drug delivery systems; combining predictive modelling with real-time monitoring (Sabbagh et al., 2025). Despite these advantages, high costs, limited scalability and unclear regulations are slowing wider adoption of these systems. Several innovations in bioprinting and 3D printing have progressed beyond preliminary research with clear differences in commercial readiness and future translational outlook (Vijayavenkataraman, 2023). Poieskin® from Poietis represents the most advanced example where a bioprinted autologous full thickness skin model has been commercialised solely for laboratory research with ongoing clinical development (Abellan Lopez et al., 2023). Similarly, L’Oreal’s bioprinted skin models have reached a stable research phase and has seen routine use for cosmetics and chemical testing; an alternative to animal testing (Girard et al., 2024). The dual-layered model has the ability to replicate the complexity of real human skin, including skin conditions, ability to heal from injury and also to tan. Full thickness skin constructs developed by Wake Forest Institute remains in the pre-clinical phase, however, there has been rapid progress in vascularisation strategies which suggests movement towards early phase clinical evaluation once reproducibility can be addressed (Jorgensen et al., 2025). At present, there are no dedicated regulations for 4D bioprinted skin-based drug delivery systems. Regulation evaluation relies on existing frameworks that govern medical devices, combination products or advanced therapy medical products, depending on material, cellular composition and intended clinical use (Demoly et al., 2021). Despite this, increased understanding of the regulations associated with 3D printed medical products, prior approval of constituent biomaterials and advances in standardisation and quality control can be expected to facilitate clinical translation of 4D bioprinting technologies in the near future. Future work in this remit depends on close collaboration between material scientists, bioengineers and regulators and should focus on combining 4D models with artificial intelligence and machine learning to improve prediction accuracy (Damiati et al., 2025; Pugliese and Regondi, 2022). Linking these systems with microfluidic platforms and imaging tools will allow non-invasive, real-time monitoring. New stimuli like biochemical gradients, circadian rhythms and immune response models could make these 4D systems more physiologically relevant.

In conclusion, while technical and regulatory challenges remain, combining 4D modelling with smart biomaterials, AI-driven analytics and integrated monitoring can reshape transdermal drug delivery. By enabling personalised, efficient, ethically responsible drug delivery strategies, 4D bioprinting and modelling represents a paradigm shift that could redefine the future of pharmaceutical development.
